# Triage and flow management in sepsis

**DOI:** 10.1186/s12245-019-0252-9

**Published:** 2019-11-21

**Authors:** Hudson Henrique Gomes Pires, Fábio Fernandes Neves, Antonio Pazin-Filho

**Affiliations:** 1 Department of Internal Medicine, Urgency and Emergency Discipline, Triangulo Mineiro Medical School, Federal University of Triangulo Mineiro, Avenida Getúlio Guaritá, 159, Bairro, Nossa Senhora da Abadia, Uberaba, Minas Gerais 38025-440 Brazil; 2 Department of Internal Medicine, São Carlos Medical School, Federal University of São Carlos, São Carlos, Brazil; 3 Department of Internal Medicine, Ribeirao Preto Medical School, University of Sao Paulo, São Paulo, Brazil

**Keywords:** Sepsis, Health care rationing, Triage, Healthcare systems, Clinical pathways

## Abstract

**Background:**

Sepsis is a major public health problem, with a growing incidence and mortality rates still close to 30% in severe cases. The speed and adequacy of the treatment administered in the first hours of sepsis, particularly access to intensive care, are important to reduce mortality. This study compared the triage strategies and intensive care rationing between septic patients and patients with other indications of intensive care. This study included all patients with signs for intensive care, enrolled in the intensive care management system of a Brazilian tertiary public emergency hospital, from January 1, 2010, to December 31, 2016. The intensivist periodically evaluated the requests, prioritizing them according to a semi-quantitative scale. Demographic data, Charlson Comorbidity Index (CCI), Sequential Organ Failure Assessment (SOFA), and quick SOFA (qSOFA), as well as surgical interventions, were used as possible confounding factors in the construction of incremental logistic regression models for prioritization and admission to intensive care outcomes.

**Results:**

The study analyzed 9195 ICU requests; septic patients accounted for 1076 cases (11.7%), 293 (27.2%) of which were regarded as priority 1. Priority 1 septic patients were more frequently hospitalized in the ICU than nonseptic patients (52.2% vs. 34.9%, *p* <  0.01). Septic patients waited longer for the vacancy, with a median delay time of 43.9 h (interquartile range 18.2–108.0), whereas nonseptic patients waited 32.5 h (interquartile range 11.5–75.8)—*p* <  0.01. Overall mortality was significantly higher in the septic group than in the group of patients with other indications for intensive care (72.3% vs. 39.8%, *p* <  0.01). This trend became more evident after the multivariate analysis, and the mortality odds ratio was almost three times higher in septic patients (2.7, 2.3–3.1).

**Conclusion:**

Septic patients had a lower priority for ICU admission and longer waiting times for an ICU vacancy than patients with other critical conditions. Overall, this implied a 2.7-fold increased risk of mortality in septic patients.

## Background

Sepsis is a major public health problem, with increasing incidence, which affects millions of people worldwide. Although the mortality rate has decreased in recent years, it remains around 30% in cases of severe sepsis [1].

Septic patients are critical since their organ systems are often unstable and require advanced support, such as mechanical ventilation, hemodialysis, and vasoactive drugs, which, ideally, should be provided in an intensive care setting [2].

The speed and adequacy of the treatment administered in the first hours of sepsis have a direct association with the outcome [3]. A multicenter study involving more than 49,000 patients found out that the delay in the initial treatment was associated with an increase in the risk of death of 4% per hour of treatment delay [4].

Initiatives were aiming to organize healthcare in the emergency room, through the development of protocols and training, which have been playing a major role in reducing sepsis mortality [5–7].

However, the fact that the emergency departments in Brazil are crowded and poorly equipped may delay the initiation of adequate therapy. Furthermore, there is a shortage of intensive care unit (ICU) beds which implicate the need for rationing and further delay the access to ICU [8, 9].

Therefore, it is necessary to develop screening mechanisms that prioritize patients who would potentially benefit more from intensive care [10–12]. Despite the unquestionable importance of triage to the rationing of intensive care resources, it is a complex and poorly standardized process [13]. Furthermore, no conclusive studies evaluated the rationing process for ICU, including sepsis.

This study aims to compare the screening strategies and allocation of ICU resources between septic patients and patients with other indications of intensive care.

## Materials and methods

The Research Ethics Committee of the Clinical Hospital of the Ribeirão Preto Medical School of the University of Sao Paulo approved the study.

### Settings and study design

This study is a retrospective cohort study of patients whose physicians included a request for a vacancy in an intensive care unit (ICU). Our institution is a tertiary hospital with 192 beds dedicated exclusively to emergency and counts with 28 ICU beds, covering a population of 4,500,000 inhabitants.

### Inclusion and exclusion criteria

We enrolled all patients with an indication for intensive care in the intensive care management system of our hospital from January 1, 2010, to December 31, 2016. We restricted the study to this period due to other flow management strategies that began in 2017.

We excluded patients/individuals whose data were incomplete on the vacancy request form and individuals under 12 years of age from the study. We included only the first request for ICU, excluding patients in need of readmission.

### Data collection and definitions

The vacancy request for intensive care at the Clinical Hospital of the Ribeirão Preto Medical School must be filed exclusively through the intensive care unit management system.

The process consists of the completion of an electronic form by the responsible physician with information about the clinical conditions of the patient, such as vital signs, urine output, Glasgow Coma Scale, and need for mechanical ventilation or vasoactive amines. Moreover, there is an indication for intensive care, with pre-defined clinical pathways (sepsis, coronary syndrome, cerebral vascular accident, and trauma), as well as the inclusion of the primary and secondary diagnoses, organized by ICD-10-CM. We did not review the sepsis and the other indications of intensive care filled by the responsible physician.

The intensivist physician periodically evaluates all cases and prioritizes them according to the following semi-quantitative scale [14]. This scale goes from priority 1, with good prognosis (severely ill patients who require mechanical ventilation or invasive hemodynamic monitoring), up to priority 4, the worst prognosis, including candidates for palliative care. Priority 2 includes patients requiring continuous monitoring and possible immediate intervention. Although they have comorbidities, investment is unlimited, since the actual acute event has a good prognosis. Priority 3 includes patients severely ill, but with a low possibility of recovery due to the underlying disease or characteristics of the acute event.

After classification, the patients are ordered by the system according to the priority given by the intensivist. In the case of a tie, the system respects the chronological order of inclusion. The intensivist physician and the hospital medical director can change the patient priority if it is necessary to eliminate potential bottlenecks to the specific flow of patients.

We retrieved demographic data, such as age, gender, ethnicity, and civil status, from the hospital information system. The characterization of surgical procedure, as well as the calculations of the Charlson Comorbidity Index (CCI), Sequential Organ Failure Assessment (SOFA), and quick SOFA (qSOFA) scores, was obtained by reviewing the electronic medical records [15, 16].

### Statistical analysis

We used mean and standard deviation or percentage for descriptive statistics according to the nature of the variable. Student’s *t* test or analysis of variance (ANOVA) or the non-parametric equivalents were used to compare continuous variables, and the chi-square test was used to compare categorical variables. For the multivariate analysis, logistic and Poisson regression models were constructed to adjust possible confounding variables regarding the outcome. All analyses were performed using the STATA software version 10 (USA, College Station TX), with a significance level below 5%.

## Results

During the study period, there were 9775 ICU vacancy requests. Since we excluded 496 observations due to the lack of evaluation by the intensivist (triage) and 84 for incomplete data in the electronic form, a total of 9195 cases remained for analysis. We classified 1076(11.7%) as septic patients.

**Fig. 1:**
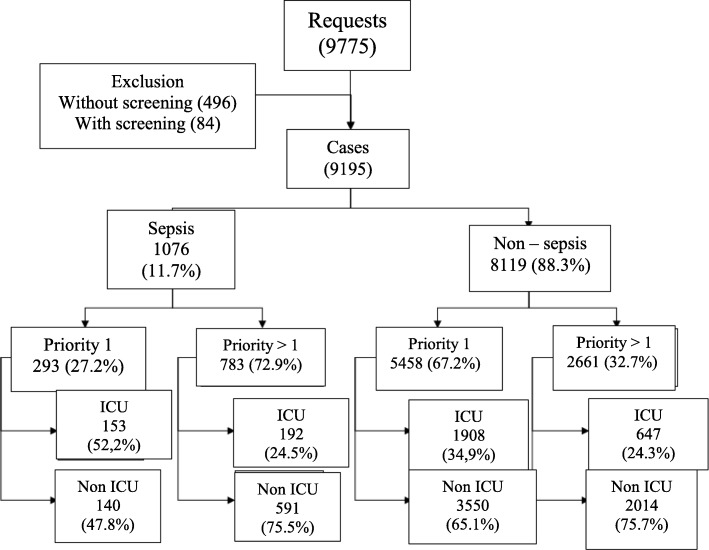
Descriptive analysis of the data of septic and nonseptic groups

Intensive care physicians classified septic patients as priority 1 in 27.2% of the cases in comparison with 67.2% for the nonseptic group (*p* <  0.01). Septic patients who received a priority 1 status had a higher proportion of ICU admission (52.2%) in comparison with nonseptic patients (34.9%) with the same classification (*p* <  0.01). Figure 1 summarizes the distribution of patients by priority 1 status and ICU admission.

Table 1 compares the demographic data and severity indicators between septic and nonseptic patients. Caucasian patients and the absence of a stable union prevailed in both groups of the male gender. Internal medicine was predominant in both groups, despite being more prevalent in the septic group (63.3% vs. 54.2%, *p* <  0.01).

**Table 1:** Descriptive analysis of the variables of septic and nonseptic patients

	Sepsis (*n* = 1076; 11.7%)	Other causes (*n* = 8119; 88.3%)	*p*
Age	59.3 ± 17.8	56.0 ± 18.2	< 0.01
Male	602 (55.9%)	5030 (61.9%)	< 0.01
Internal medicine specialty	682 (63.4%)	4405 (54.2%)	< 0.01
Married/stable union	411 (38.2%)	3358 (41.3%)	0.04
Declared Caucasian ethnicity	856 (79.5%)	6358 (78.3%)	0.35
Charlson score ≥ 1	699 (64.9%)	4517 (55.6%)	< 0.01
qSOFA score ≥ 2	639 (59.3%)	3641 (44.8%)	< 0.01
SOFA score ≥ 2	822 (76.4%)	5686 (70.0%)	< 0.01
Vasoactive drugs	788 (73.2%)	1988 (24.5%)	< 0.01
Mechanical ventilation	693 (64.4%)	3758 (46.3%)	< 0.01

Patients diagnosed with sepsis were older and had more comorbidities than the group of patients with other indications of intensive care. They also had a higher prevalence of organ dysfunction and, therefore, required more intensive care resources, such as mechanical ventilation and infusion of vasoactive drugs.

Despite the significant differences between the groups, both in terms of comorbidities and severity, the proportion of access to intensive care was similar; 345 of the 1076 septic patients (32.0%) were admitted to the ICU, whereas 2555 of the 8119 nonseptic patients (31.4%) were referred to the ICU (*p* = 0.31).

Even though there was a similar frequency of ICU admission between groups, septic patients waited longer for the vacancy, with a median time delay of 43.9 h (interquartile range 18.2–108.0), whereas nonseptic patients waited 32.5 h (interquartile range 11.5–75.8)—*p* < 0.01.

Overall mortality was significantly higher in the septic group than in the group of patients with other indications for intensive care (72.3% vs. 39.8%, *p* < 0.01). This trend became more evident after the multivariate analysis, and death risk was almost three times higher in septic patients (OR 2.7, 2.3–3.1).

**Fig. 2:**
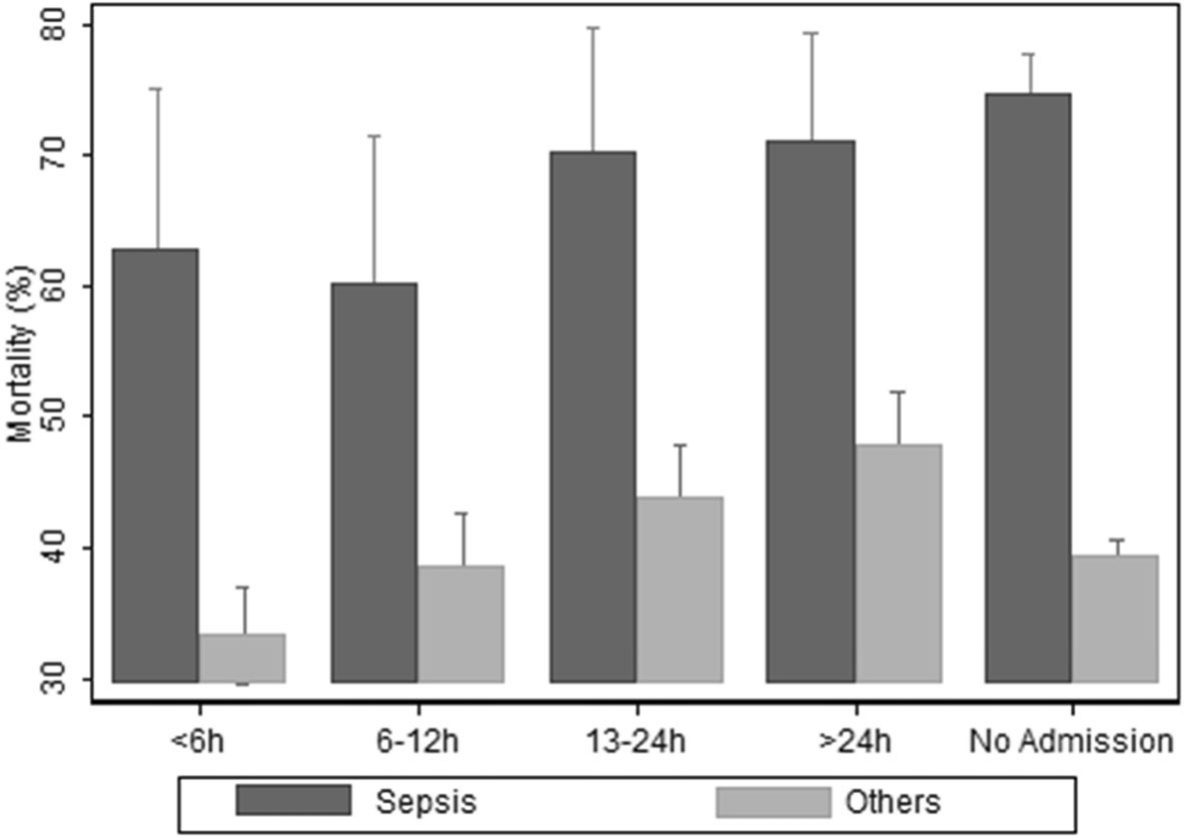
Analysis of mortality associated with ICU admission delay in hours between septic and nonseptic patients

The delay in ICU admission directly affected this outcome. Figure 2 shows that mortality in nonseptic patients increased in a linear manner according to the magnitude of the delay in intensive care admission, starting at 33.3% on average in patients who had been admitted in less than 6 h and reaching 47.8% in those whose admission was delayed for more than 24 h (*p* for trend < 0.01). We observed two mortality patterns in the group of septic patients. Those patients with up to 12 h delay reached 60% mortality, while those with more than 12 h had a 70% mortality prevalence (*p* = 0.057). Septic patients admitted for more than 12 h had the same mortality rate as septic patients that had not been admitted (*p* = 0.87).

**Fig. 3:**
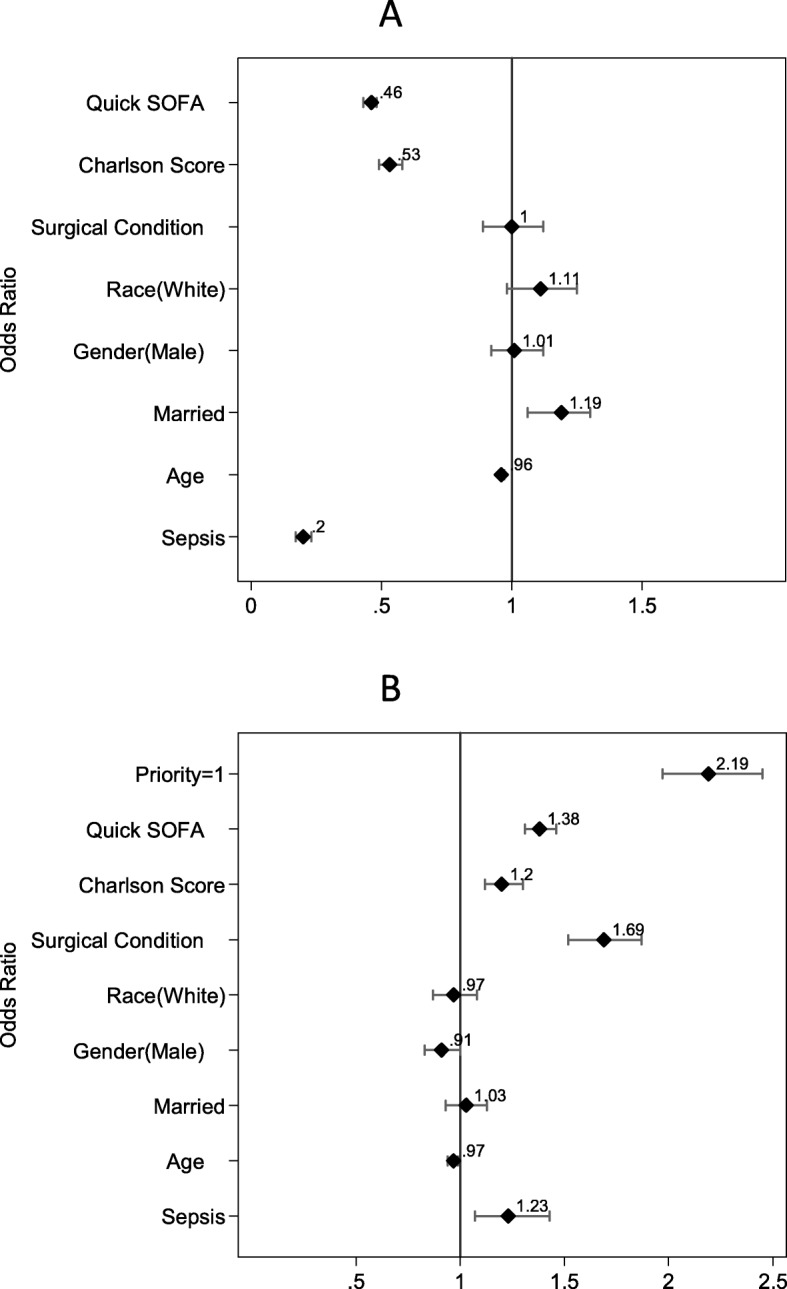
OR (odds ratio) to admitting priority 1 (**a**) and to being admitted to ICU (**b**), according to the variable analyzed

In the multivariate analysis (Fig. 3a), sepsis was the independent variable more negatively associated with receiving a priority 1 classification during medical screening. Sepsis was even more significant than the presence of comorbidities (Charlson Score), with odds ratios of 0.20 (0.17–0.23) and 0.53 (0.49–0.58), respectively.

On the other hand, despite being regarded as low priority by intensive care physicians, septic patients were more likely to be admitted to intensive care, second only to surgical patients, with odds ratio of 1.23 (1.07–1.43) and 1.69 (1.52–1.87)—Fig. 3b.

## Discussion

Septic patients had a lower priority for ICU admission and longer waiting times for an ICU vacancy than patients with other critical conditions, implying a 2.7-fold increased risk of mortality in septic patients. The delay in ICU admission was associated with a higher mortality rate in both groups, with a linear behavior in the nonseptic group and bimodal trend in the septic group.

The mortality rate observed in the septic group (73.3%) was higher than in other countries with limited resources, such as Haiti—24.2% [17], Uganda—45.7% [18], Colombia—33.6% [19], and several Asian countries—44.5% [20]. A Brazilian study which analyzed data from 4271 hospitals over 10 years estimated the overall sepsis mortality at 46.3%. In the group of patients that required ICU transfer, the mortality rate was 64.5% [21]. A prevalence study conducted by Machado et al. in 1690 Brazilian intensive care units found 30% of intensive care beds occupied by septic patients [22]. The 55.7% mortality rate found in this population was lower than the observed in this study, even in patients who also needed intensive care resources.

Given the number of confounders involved, it is difficult to compare the mortality of septic patients in different populations. The sepsis definition has undergone revisions, requiring caution when comparing studies. Much remains to be done so that sepsis is recognized early. Finally, the conditions of access to the healthcare system and its structuring are significant points to consider, since sepsis is a time-sensitive condition.

Papali et al. proposed the three-delay model, in which barriers are used to effectively evaluate the treatment of septic patients, especially in countries with limited resources [23]. These barriers are recognizing and diagnosing sepsis at admission (first), providing expedite initial resuscitation measures (second), and providing follow-up and monitoring after clinical resuscitation (third). The first two barriers are closely associated with the quality of care at the emergency department, whereas the latter depends on the timely qualified provision of intensive care beds.

We observed that less than one third of septic patients with indications for intensive care in the present study were admitted to the ICU, and most of them ended up receiving care at the emergency department, often without the resources needed. The unavailability of ICU beds is a global problem that requires urgent structural investments, as well as the adoption of rationing strategies to limited resources [12, 13, 24].

Three models of rationing ICU admission have been discussed: prioritization, list of diagnoses, and vital parameters [3]. The prioritization is a process that utilizes a semi-qualitative scale standardizing the benefit that admission to the ICU could bring to the patient. Meanwhile, the diagnostic list and vital parameter models seek to grant more objectivity to the evaluation. The first model was based on a list of diagnoses that should be prioritized, and the second depends on the patient’s functional condition, that is, the degree of instability. Even though more objective models may be more attractive, more recent evidence suggests the use of semi-qualitative models, like the one used in this study [3].

The results obtained herein reinforce the importance of providing timely access to intensive care resources. The association between ICU transfer delay and increased in-hospital mortality, both for septic patients and patients with other conditions, that we observed is similar to those of other researchers [9, 25–27]. Administrative protocols implementation that prioritizes the admission of septic patients to intensive care resources could be a valuable tool to reduce mortality. These protocols proved to be worthy in other time-sensitive diseases, such as acute coronary syndromes and cerebral vascular accident.

The worst prognosis associated with out-of-ICU care may be due to the usual overcrowding of emergency departments and the consequent lack of structural and human resources, thus hindering the proper development of the best clinical practices. A retrospective cohort of septic patients demonstrated that fluid infusion and antibiotic administration delayed progressively as the occupancy rate increased in the emergency department [28]. Another potential explanation is the lack of training of the health care personnel of the emergency departments and nursing wards to deal with critically ill patients [9].

The lack of validated criteria to screen a septic patient for ICU admission is a complicating factor. In this study, only 27.2% of septic patients were classified as priority 1, whereas in the nonseptic group, 67.2% received this classification. Even though we have not measured interobserver variability, a hierarchical superior mediates any disagreement in our rationing process. Nevertheless, this process could not rule out any preconceived notion of a lower prognosis of sepsis that more objective screening criteria could aid to reduce. In a study conducted in Brazil, Cardoso et al. observed that sepsis was the only diagnostic category significantly associated with ICU admission delay [9].

Older age and higher prevalence of comorbidities in the septic patient group could be responsible for their worst classification, and consequently, with less potential benefit from intensive care resources. Quintano et al. concluded that age is an independent predictor of death in sepsis and that there is an additional risk of dying of 3.6% per year of life [21]. As for comorbidities, Cardoso et al. observed a higher prevalence of these conditions in the group of patients with delayed ICU admission when compared to patients with immediate access, with prevalence of 25.4% and 10.4%, respectively [9].

The increased prevalence of septic patients referred from internal medicine may also help to understand the logic used in screening. Internist doctors are better trained to care for critical patients than surgeons, which could lead intensivists to select patients from surgical specialties.

Paradoxically, priority 1 septic patients were admitted to the ICU more frequently than nonseptic patients. This finding may be explained by the increased severity in septic patients and by a higher prevalence of organ dysfunction. The higher prevalence of organ dysfunction among septic patients, making it difficult for them to stay in non-specialized beds, could explain this finding.

Some considerations should be made regarding the distinct behavior when associating mortality rate with ICU admission delay between septic (bimodal) and nonseptic (linear) patients. It should be noted that the time frame for the onset of clinical presentation of nonseptic conditions such as trauma, infarction, and cerebral vascular accident is easier to characterize than that of sepsis. As the septic patients included in this study were referred from other hospital institutions, it was not possible to define a group in which the onset of sepsis was better delimited. Since the therapeutic interventions in the septic and nonseptic groups are associated with the temporal window of opportunity, any treatment that the septic patients received may have occurred relatively late.

The bimodal behavior of ICU admission delay and mortality in septic patients reinforces the need to foster awareness of the concept of sepsis and early onset of treatment. Physicians undervalue the most readily available therapeutic interventions for sepsis (hydration and antibiotics) in comparison with those for other time-sensitive conditions, such as thrombolytics or surgical interventions. Taking the structuring of the healthcare system referral into account, we should promote the adoption of these interventions in the prehospital environment.

Some limitations have been observed in this study. Firstly, data from a single center were analyzed, thus determining a low external validity. Secondly, due to the retrospective design of our research, we could not rule out selection biases. We could not revise the conditions that led to an ICU admission request and the classification of the groups according to diagnostic criteria established by international guidelines. Thirdly, the nonseptic patients could be heterogeneous and make the comparison with septic patients unfair. Nevertheless, the lack of ICU beds granted that the majority of the nonseptic group was composed of time-sensitive conditions (trauma, acute coronary syndromes, and central nervous system vascular conditions). We made the comparison of the septic group in isolation with each condition and found out no significant difference and decided to keep the results in only two groups for the sake of simplicity. Finally, due to the high heterogeneity of data described in the electronic medical records, it was not possible to apply more powerful prognostic indicators. Such limitations require careful interpretation of our findings.

## Conclusions

Septic patients received a lower priority of access to intensive care during the triage process by intensivist in our institution. This unfavorable evaluation made the sepsis victims expect more for a vacancy in the ICU compared to patients with other critical conditions. The delay in ICU admission was associated with higher mortality in both groups. Mortality risk was 2.7 times higher in septic patients. Administrative protocols that prioritize the access of septic patients to intensive care resources like those used in other time-sensitive diseases could be of benefit.

## Data Availability

Please contact the authors for data requests.
